# Association of True Positivity with Serum Prostate-Specific Antigen Levels and Other Clinical Factors in Indeterminate PSMA-RADS-3A Lesions Identified on ^18^F-DCFPyL PET/CT Scans

**DOI:** 10.3390/tomography8060220

**Published:** 2022-10-27

**Authors:** Tushar Garg, Rudolf A. Werner, Hyun Woo Chung, Wajahat Khatri, Kenneth J. Pienta, Martin G. Pomper, Michael A. Gorin, Elie Saad, Steven P. Rowe

**Affiliations:** 1 The Russell H. Morgan Department of Radiology and Radiological Science, Johns Hopkins University School of Medicine, Baltimore, MD 21287, USA; 2 Department of Nuclear Medicine, University Hospital Würzbürg, 97080 Würzburg, Germany; 3 Department of Nuclear Medicine, Konkuk University Medical Center, Konkuk University School of Medicine, Seoul 05030, Korea; 4 Department of Radiology, Harlem Hospital, New York, NY 10037, USA; 5 Department of Urology, The James Buchanan Brady Urological Institute, Johns Hopkins University School of Medicine, Baltimore, MD 21287, USA; 6 The Milton and Carroll Petrie Department of Urology, Mount Sinai Health System, New York, NY 10029, USA

**Keywords:** prostate cancer, prostate-specific antigen, PSMA-RADS, ^18^F-DCFPyL PET/CT, Gleason score

## Abstract

The use of prostate-specific membrane antigen targeted PET imaging for the evaluation of prostate cancer has increased significantly in the last couple of decades. When evaluating these imaging findings based on the PSMA reporting and data system version 1.0, which categorize lesions based on their likelihood of prostate cancer involvement, PSMA-RADS-3A lesions are commonly seen, which are indeterminate for the presence of disease. A total of 28 patients with 171 PSMA-RADS-3A lesions on ^18^F-DCFPyL PET/CT scans from June 2016 to May 2017 who had follow-up cross-sectional imaging over time were included in this study. The PSA levels of patients with PSMA-RADS-3A lesions were categorized into four groups, 0–0.2, 0.2–1, 1–2, and >2 ng/mL. The pre-operative Gleason score of these patients was categorized into two groups, Gleason score < 7 or ≥7. The median age for these patients was 72.5 years (range 59–81). The median PSA value for patients with positive lesions was significantly higher than those with negative lesions (5.8 ng/mL vs. 0.2 ng/mL, *p* < 0.0001). The lesion positivity rate was significantly higher in patients with PSA > 1 ng/mL (18.2% vs. 81.9%, *p* < 0.001). On ROC analysis, the highest classification accuracy was seen at PSA ≥ 0.6 ng/mL of 80.12% (95% CI = 73.69–86.16%), and the area under the curve was 71.32% (95% CI = 61.9–80.7%, *p* < 0.0001). A total of 96.4% (108/112) of patients with positive lesions and 86.4% (51/59) of patients with negative lesions had a PSMA-RADS-4/5 lymph node on the initial ^18^F-DCFPyL PET/CT scan (*p* = 0.02). In patients with a Gleason score ≥ 7, the presence of positive PSMA-RADS-3A lesions was higher, compared to negative PSMA-RADS-3A lesions (*p* = 0.049). Higher PSA levels in patients with PSMA-RADS-3A lesions can point towards the presence of true positivity. PSA levels may be considered in deciding whether to call an indeterminate lesion on PSMA PET.

## 1. Introduction

Prostate cancer is the second most commonly occurring cancer, representing 14% of all new cancer cases in the United States [1]. In 2019, the age-adjusted incidence rate of prostate cancer diagnosis was 123.1 per 100,000 individuals, according to the Surveillance, Epidemiology, and End-Results (SEER) database [2]. Multiple imaging modalities are used for imaging men with prostate cancer, such as transrectal ultrasound (TRUS), magnetic resonance imaging (MRI), computed tomography (CT), ^99m^Tc-methylene diphosphonate bone scan, and positron emission tomography (PET). PET is now commonly used to identify sites affected by prostate cancer on initial staging and subsequent follow-up. PET is combined with CT into one hybrid imaging system in today’s scanners, and patients can be scanned using a variety of radiopharmaceuticals. Available radiopharmaceuticals for PET/CT include 2-deoxy-2-[^18^F]fluoro-D-glucose, ^18^F-NaF, ^11^C-choline, ^18^F-fluciclovine, and the prostate-specific membrane antigen (PSMA)-targeted class of agents. The PMSA-targeted agents (such as ^18^F-DCFPyL) have shown high sensitivity and specificity for the detection of local and distant metastatic lesions of prostate cancer, compared to conventional cross-sectional imaging modalities and other classes of radiopharmaceuticals [3,4,5,6,7,8,9,10].

The ^18^F-DCFPyL PET/CT has been used extensively in staging patients prior to surgery if they are at risk for pelvic lymph node involvement, evaluation of patients found to have biochemical recurrence after definitive therapy, and evaluation of patients with oligometastatic prostate cancer (≤5 sites of distant disease) [3,4,11]. The radiotracers targeting PSMA can show variable uptake in sites affected by prostate cancer and show uptake in non-prostate malignancies, as well as benign lesions [12,13,14,15]. In 2018, Rowe et al. proposed a structured reporting system called the PSMA reporting and data system (PSMA-RADS) version 1.0 to report the findings of PSMA-targeted PET studies, which has shown a high concordance rate amongst readers with different levels of experience [16,17]. In this reporting system, lesions involving the lymph nodes or other soft tissues that need further workup or follow-up imaging to completely characterize the findings are classified as PSMA-RADS-3A [17]. Previous studies have reported that approximately 75% of lesions categorized as PSMA-RADS-3A will eventually be declared as metastatic sites for prostate cancer on follow-up imaging [18]. In a follow-up study by the same group, point-spread function reconstruction was used to evaluate PSMA-RADS-3A lesions, which allowed the re-categorization of 7.6% of PSMA-RADS-3A lesions into PSMA-RADS-4 [19]. 

The true positivity rate of PSMA-RADS-3A sites detected on ^18^F-DCFPyL PET/CT correlated with different PSA values has not been evaluated. As such, the aim of this study was to investigate the role of contemporaneous PSA values on the positivity of PSMA-RADS-3A lesions detected on ^18^F-DCFPyL PET/CT performed for evaluating biochemical recurrence of prostate cancer, oligometastatic prostate cancer, or initial staging of prostate cancer.

## 2. Materials and Methods

### 2.1. Patient Recruitment

A total of 275 consecutive patients who underwent ^18^F-DCFPyL PET/CT scans between June 2016 and May 2017 as a part of the diagnostic evaluation of their pathologically diagnosed prostate cancer were included in the initial screening for this institutional review board approved, *post hoc* analysis of a prospective study (NCT02825875). All the patients in this prospective study were scanned under a US Food and Drug Administration Investigational New Drug Application (IND 121064), prior to the regulatory approval of ^18^F-DCFPyL in May 2021. Informed consent was obtained from all patients in the original trial. After reviewing the ^18^F-DCFPyL PET/CT scans for these patients, all patients (n = 28) with at least one PSMA-RADS-3A lesion were identified and included in the final analysis. 

All procedures performed in studies involving human participants were in accordance with the ethical standards of the institutional and national research committee and with the 1964 Helsinki Declaration and its later amendments or comparable ethical standards. 

### 2.2. Data Collection

The electronic medical records of the patients with at least one PSMA-RADS-3A lesion were reviewed retrospectively to collect information about age, absolute prostate-specific antigen (PSA) level before the ^18^F-DCFPyL PET/CT scan, original Gleason score during the time of prostate cancer diagnosis, and treatment modalities used before and after ^18^F-DCFPyL PET/CT scan for management of prostate cancer.

### 2.3. Image Acquisition

The radiosynthesis of ^18^F-DCFPyL was performed similarly to the previously described method by Ravert et al. [20]. The acquisition protocol for the images has been described in detail previously [12]. In brief, the patients were not allowed to eat or drink for 6 h prior to radiotracer administration. A dose of ≤333 MBq (≤9 mCi) of ^18^F-DCFPyL was then administered via slow IV push, and the patient was asked to void urine 1 h after the injection before being placed supine on the scanner for imaging. The scans were performed using whole-body PET/CT clinical scanners (128-slice Biograph mCT scanner [Siemens Healthineers, Erlangen, Germany] or 64-slice DVCT [GE Healthcare, Waukesha, WI, USA]). The images obtained from the scanners were reconstructed with standard ordered subset expectation maximization (OSEM) algorithms supplied by the manufacturers. 

### 2.4. Image Analysis

**Figure 1:**
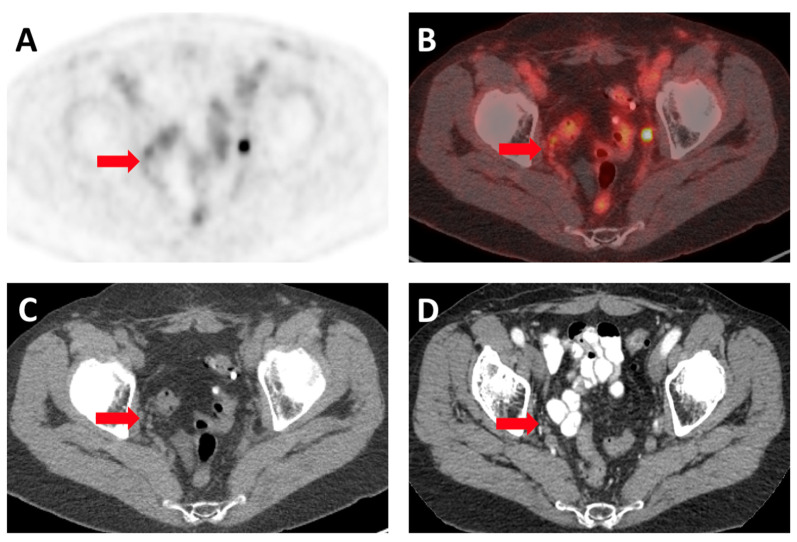
Images from a 72-year-old man being evaluated for biochemical recurrence of prostate cancer with PSA 1.2 at the time of imaging. (**A**) Axial ^18^F-DCFPyL PET and (**B**) PET/CT images. There is a small right obturator fossa lymph node with faint uptake just posterolateral to more intense uptake in the right ureter (arrows). (**C**) Attenuation-correction CT image from the ^18^F-DCFPyL PET and (**D**) diagnostic CT image from 6 months after the patient was started on androgen deprivation therapy. The lymph node has nearly disappeared on follow-up (arrows), suggesting true positivity.

^18^F-DCFPyL PET/CT scans were reviewed and characterized according to the PSMA-RADS version 1.0 by an experienced reader (HWC) [17]. The findings were verified by another experienced reader (SPR) Briefly, PSMA-RADS-3A was assigned to lesions with equivocal uptake in soft-tissue sites (all lymph nodes in this cohort) that are typically involved in prostate cancer. In patients with advanced disease, PSMA-RADS-3A was assigned to sites outside those regularly involved by prostate cancer, such as the mediastinum and left-supraclavicular space. For patients with PSMA-RADS-3A lesions, if any follow-up cross-sectional imaging was available, it was reviewed in order to ascertain if these lesions had developed into true disease or not. The following criteria were used as a standard of truth to determine if the initial PSMA-RADS-3A lesions were true positive for prostate cancer (adapted from Yin et al.) [18]:

1. Follow-up ^18^F-DCFPyL PET/CT scan showing significantly increased or decreased uptake of the radiotracer in the PSMA-RADS-3A lesion signified by a SUV_max_ change of greater than 30%.
2. Follow-up CT/MRI scan showing more than 2 mm increase or decrease in the diameters of the PSMA-RADS-3A lesions (Figure 1).

### 2.5. Statistical Analysis

The patient demographics and clinical information were reported as medians with ranges or proportions, as deemed appropriate. The PSA levels of patients with PSMA-RADS-3A lesions were categorized into four groups, 0–0.2, 0.2–1, 1–2, and >2 ng/mL. These groups were compared to see if the rates of positivity differed based on the PSA value using the chi-squared test. Additionally, the PSA values of true positive or true negative PSMA-RADS-3A lesions were compared using the Mann–Whitney test. The pre-operative Gleason score of these patients was categorized into two groups, Gleason score < 7 or ≥7, and the rate of positivity of PSMA-RADS-3A lesions was compared using the chi-squared test. The presence of PSMA-RADS-4/5 lesions and the rate of PSMA-RADS-3A lesion true positivity were compared using Fischer’s exact test. Finally, an ROC analysis was performed using the PSA values to identify the cut-off point for PSA with the highest accuracy to predict the true positivity of PSMA-RADS-3A lesions.

## 3. Results

A total of 275 patients with available clinical and imaging parameters were screened. Out of these 275 patients, 89 (32.3%) of them were found to have at least one PSMA-RADS-3A lesion on their ^18^F-DCFPyL PET/CT scans. Only 31.5% (28/89) patients had appropriate cross-sectional imaging follow-up available to evaluate for changes in PSMA-RADS-3A lesions over time. Therefore, those 28 patients were included in the final analysis. The median age for those patients was 72.5 years (range 59–81), with 71.4% (20/28) undergoing ^18^F-DCFPyL PET/CT scan for evaluation of biochemical recurrence or PSA persistence after local therapy, 14.3% (4/28) for the initial staging of prostate cancer and 14.3% (4/28) for the evaluation of metastatic disease. The most common pre-^18^F-DCFPyL PET/CT scan therapy was radical prostatectomy in 82.1% (23/28) of patients, followed by radiation therapy in 42.9% (12/28) of patients, and androgen deprivation therapy in 21.4% (6/28) of patients (Table 1). The most common post-^18^F-DCFPyL PET/CT scan therapy was androgen deprivation therapy in 71.4% (20/28) of patients, followed by radiation therapy in 50% (14/28) of patients, and chemotherapy in 32.1% (9/28) of patients (Table 1).

**Figure 2:**
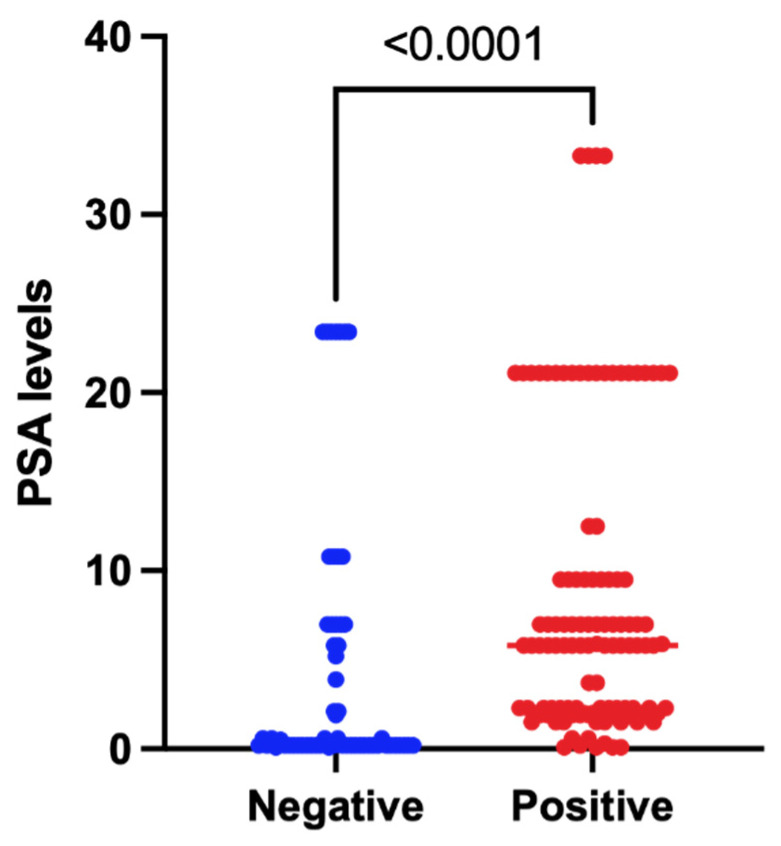
Comparison of pre-^18^F-DCFPyL PET/CT scan PSA levels in positive and negative PSMA-RADS-3A lesions on follow-up.

A total of 171 PSMA-RADS-3A lesions were identified across 28 patients, of which 65.5% (112/171) of these were found to be positive based on follow-up imaging. The overall median PSA value for these patients was 3.0 (0.1–33.3) ng/mL. On lesion level analysis, the median PSA value for patients with positive lesions was 5.8 (0.1–33.3) ng/mL and for patients with negative lesions was 0.2 (0.1–23.4) ng/mL. The PSA values for patients with positive lesions were significantly higher, with an actual difference of 5.6 ng/mL, and a Hodges–Lehmann difference of 2.1 ng/mL (*p* < 0.0001) (Figure 2).

**Figure 3:**
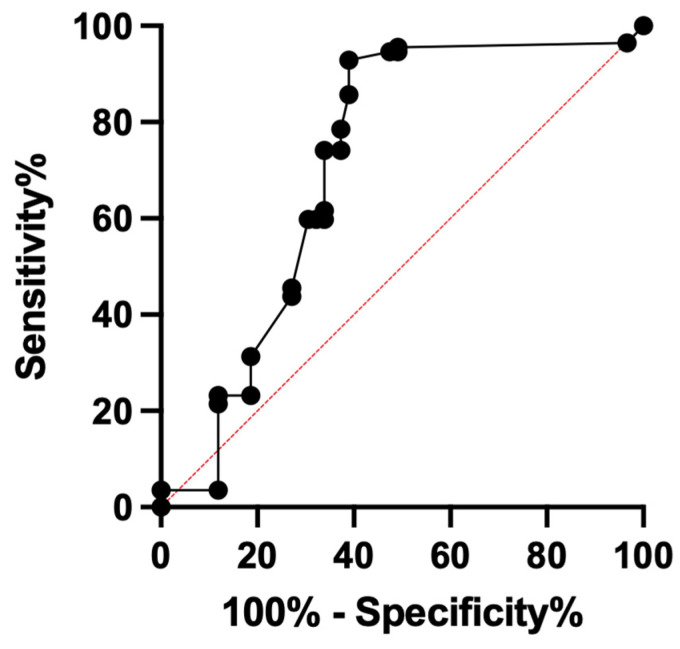
ROC curve of contemporaneous PSA levels in predicting positive PSMA-RADS-3A lesions on follow-up.

When looking at the different PSA categories, 35 lesions were in patients with PSAs of 0.0–0.2 ng/mL, 9 lesions in patients with PSA 0.2–1.0 ng/mL, 22 lesions in patients with PSA 1.0–2.0 ng/mL, and 105 lesions in patients with PSA > 2.0 ng/mL category. A total of 16.6% (5/35) and 33.3% (3/9) of the lesions in 0.0–0.2 ng/mL and 0.2–1.0 ng/mL categories were positive, whereas 95.5% (21/22) and 79% (83/105) lesions in the 1.0–2.0 ng/mL and >2.0 ng/mL categories were positive. The lesion positivity rate was different across categories (Pearson Chi^2^ = 62, *p* < 0.001) and was significantly higher in lesions from patients with PSA > 1.0 ng/mL (18.2% vs. 81.9%, *p* < 0.001). On ROC analysis, the highest classification accuracy of 80.12% (95% CI = 73.69–86.16%) was seen at PSA ≥ 0.6 ng/mL with sensitivity of 95.5% (95% CI = 88.80–98.03%) but low specificity of 50.8% (95% CI = 38.03–65.34%). The area under the curve (AUC) was 71.32% (95% CI = 61.9–80.7%, *p* < 0.0001) (Figure 3).

The overall median Gleason score pre-^18^F-DCFPyL PET/CT scan was 7 (range, 6–9). Of the total 171 PSMA-RADS-3A lesions, 6.4% (11/171) were from patients with a Gleason score of < 7 and 93.6% (160/171) were from a patient with a Gleason score of ≥7. A total of 96.4% (108/112) of the positive lesions were from patients with a Gleason score ≥ 7, compared to 88.13% (52/59) of the negative lesions being from patients with a Gleason score > 7. In patients with a Gleason score ≥ 7, the presence of positive PSMA-RADS-3A lesions was higher compared to negative PSMA-RADS-3A lesions (*p* = 0.049). A total of 96.4% (108/112) of patients with positive lesions and 86.4% (51/59) of patients with negative lesions had a PSMA-RADS4/5 node on the ^18^F-DCFPyL PET/CT scan (*p* = 0.02).

## 4. Discussion

PSMA-targeted PET imaging is increasingly being used for the evaluation of patients with prostate cancer for staging, detecting oligometastatic sites, and evaluation of biochemical recurrence. PSMA-RADS-3A lesions, which represent indeterminate findings in lymph nodes or other soft-tissue sites, can be commonly identified on these scans. In this retrospective analysis of a prospective clinical trial, contemporaneous PSA values of patients with PSMA-RADS-3A lesions were evaluated for their association with true positivity in those lesions. PSA value >1.0 ng/mL was seen in 81.9% of patients with true positive PSMA-RADS-3A lesions, and a PSA value ≥ 0.6 ng/mL was found to have an accuracy of 80.1% in correctly categorizing these lesions with a sensitivity of 95.5%.

PSA is a glycoprotein that is secreted by both normal and neoplastic prostate tissue. In normal patients, PSA is produced by the secretory cells that line the prostate glands in the form of a proenzyme, which is cleaved in the lumen to generate active PSA. The active PSA later undergoes proteolysis to form inactive PSA, and a small amount of this inactive PSA enters the bloodstream (unbound). In patients with prostate cancer, the relative amount of inactive PSA is lesser, compared to active PSA. However, absolute PSA (active and inactive) is generally used for determining the extent of prostate cancer, assessing response to treatment, and following up the patients for recurrence of prostate cancer. Based on the results of this study, a PSA level ≥ 0.6–1.0 ng/mL can serve as a marker for a high index of suspicion for true positivity in patients with PSMA-RADS-3A lesions. The presence of more definitive sites of prostate cancer on the ^18^F-DCFPyL PET/CT scan has also been found to have higher rates of PSMA-RADS-3A lesion positivity on follow-up [18]. The presence of a Gleason score ≥ 7 has been shown to predict biochemical recurrence of prostate cancer after radical prostatectomy, the presence of lymph node metastases, and the presence of oligometastatic disease [21,22,23]. Similarly, in our patient cohort, the presence of a Gleason score ≥ 7 was found to have a higher rate of PSMA-RADS-3A lesion positivity. Therefore, in patients with these three findings, stringent follow-up should be considered with either cross-sectional imaging at 3–6 months or tissue biopsy. For patients who may undergo metastasis-directed therapy, the factors outlined in this study that contribute to true positivity of indeterminate lesions may help guide decisions regarding the extent and distribution of therapy.

This study suffers from some limitations. Firstly, there was a lack of histopathological confirmation of PSMA-RADS-3A lesions and reliance on follow-up imaging to confirm the presence or absence of disease. Second, this study was a retrospective (post hoc) analysis of a prospective clinical trial with a relatively low sample size due to the lack of follow-up in a majority of patients who were found to have PSMA-RADS-3A lesions. This study lacks the strength of a prospectively powered clinical trial to definitely ascertain the predictors of true-positive indeterminate findings on PSMA PET. The patients included in this analysis had often had a number of prior therapies, and we are unable to provide any statistically meaningful sub-group analyses that might provide insight into true-positivity rate based on prior therapeutic approaches. Further, the various PSMA-targeted radiotracers that are in clinical use have different biodistributions, noise characteristics, and detection efficiencies, so the broad generalizability of these findings beyond ^18^F-DCFPyL is unknown. Therefore, the prospective long-term evaluation of patients with PSMA-RADS-3A lesions with histopathological confirmation is needed to address these limitations. 

## 5. Conclusions

This study provides preliminary data for the inclusion of pre-18F-DCFPyL PET/CT scan PSA values, original Gleason score, and presence of definitive sites of prostate cancer (PSMA-RADS-4/5) in the decision algorithm when deciding on further management for prostate cancer patients with PSMA-RADS-3A lesions.

## Figures and Tables

**Table 1:** Pre- and post-^18^F-DCFPyL PET/CT scan therapies for patients included in the study cohort (n = 28).

Pre-^18^F-DCFPyL PET/CT Scan Therapy	Number (%)
Radical prostatectomy	23 (82.1)
Radiation therapy	12 (42.9)
Androgen deprivation therapy	6 (21.4)
Chemotherapy	3 (10.7)
Vaccine therapy	1 (3.6)
Salvage radiation	1 (3.6)
Salvage pelvic lymph node dissection	1 (3.6)
None	3 (10.7)
**Post-^18^F-DCFPyL PET/CT scan therapy**	
Androgen deprivation therapy	20 (71.4)
Radiation therapy	14 (50)
Chemotherapy	9 (32.1)
Radical prostatectomy	2 (7.1)
Cryoablation	1 (3.6)
Salvage pelvic lymph node dissection	1 (3.6)

## Data Availability

The data presented in this study are available on request from the corresponding author. The data are not publicly available due to privacy concerns.
